# Exploitation in International Paid Surrogacy Arrangements

**DOI:** 10.1111/japp.12138

**Published:** 2015-07-14

**Authors:** Stephen Wilkinson

**Affiliations:** ^1^ Department of PoliticsPhilosophy and ReligionCounty SouthLancaster UniversityLancasterLA1 4YQUK

## Abstract

Many critics have suggested that international paid surrogacy is exploitative. Taking such concerns as its starting point, this article asks: (1) how defensible is the claim that international paid surrogacy is exploitative and what could be done to make it less exploitative? (2) In the light of the answer to (1), how strong is the case for prohibiting it? Exploitation could in principle be dealt with by improving surrogates' pay and conditions. However, doing so may exacerbate problems with consent. Foremost amongst these is the argument that surrogates from economically disadvantaged countries cannot validly consent because their background circumstances are coercive. Several versions of this argument are examined and I conclude that at least one has some merit. The article's overall conclusion is that while ethically there is something to be concerned about, paid surrogacy is in no worse a position than many other exploitative commercial transactions which take place against a backdrop of global inequality and constrained options, such as poorly‐paid and dangerous construction work. Hence, there is little reason to single surrogacy out for special condemnation. On a policy level, the case for prohibiting international commercial surrogacy is weak, despite legitimate concerns about consent and background poverty.

## 1. Introduction

… transnational commercial contract pregnancy involves the exploitation of impoverished and often uneducated Indian women and … this exploitation is morally objectionable.^1^

Many commentators have suggested that paid surrogacy, especially cross‐border or international paid surrogacy, is (or is very likely to be) exploitative.^2^ Taking such concerns as its starting point, this article addresses the following questions.

(1)  How defensible is the claim that international paid surrogacy is exploitative and what could be done to make it less exploitative?
(2)  In the light of the answer to (1), how strong is the case for prohibiting international paid surrogacy?

This article focuses just on exploitation‐related arguments. This is not, however, meant to imply that there are no other equally important critiques of international paid surrogacy. Others include those based on: harm to the children created, harm to the surrogates, adverse effects on the social position of women, and the suggestion that surrogates are wrongly ‘commodified’ or treated as mere means to others' reproductive ends.^3^

It should also be noted that while the focus here is solely on the exploitation of surrogates, commissioning couples may also be vulnerable to exploitation. This could be because they are relatively impoverished themselves and can't afford to access surrogacy services at home, because they are desperate for a child, or because they are overcharged by surrogacy agencies.^4^ In addition, exploitation by surrogates of the commissioning parent(s) is possible. In jurisdictions like the UK where surrogacy contracts are not enforceable and the gestational mother is the legal mother at birth, some surrogates have been known to refuse to cooperate with transferring legal parentage; others have defrauded the commissioning parents by faking pregnancy or miscarriage.^5^ However, while disputes between surrogates and commissioning couples sometimes receive a great deal of publicity,^6^ and are ethically interesting and important in their own right, these incidents are generally believed to be rare.^7^

Finally, as regards scope limitations, this discussion of ‘cross‐border or international’ surrogacy focuses solely on transactions involving relatively wealthy commissioning couples from Western countries and surrogates from developing world or LMIC countries: India being the leading example and the one mentioned most here. The reason for this scope limitation is that these are the cases in which exploitation concerns seem most pressing, while the reason for citing India in particular is simply the scale of the practice there, with (for example) the number of children born through surrogacy in India to UK parents possibly being as high as 1,000 per year.^8^

The article proceeds as follows. After making some preliminary points about exploitation, it goes on to argue that concerns about exploitation can in principle be dealt with just by improving surrogates' pay and conditions. However, doing so may generate or exacerbate problems with the surrogates' consents. Foremost amongst these is the argument that surrogates from economically disadvantaged countries cannot validly consent because their background poverty is coercive. Several different versions of this argument are examined and I conclude that at least one has merit. We do therefore have cause to be concerned about the quality of surrogates' consent.

The final part of the article asks what follows from this. I conclude that while ethically there is something to be concerned about here, paid surrogacy is likely to be in no worse a position than many other exploitative commercial transactions which take place against a backdrop of global inequality and constrained options, such as poorly‐paid and dangerous factory work. Hence, there is little reason to single surrogacy out for special condemnation and, on a policy level, the case for prohibiting international commercial surrogacy is weak, despite people's legitimate concerns about consent and background poverty.

## 2. Exploitation

… we can certainly distinguish two elements of exploitation – taking advantage of someone's (relative) poverty to get them to do something they would not otherwise do, and taking advantage of someone's (relative) poverty to get them to accept a price lower than the ‘fair’ price.^9^

This section contains some general observations about what it takes for an arrangement or practice to be exploitative. I do not claim to offer a full account or theory of exploitation here.^10^ Rather, I merely draw attention to some important distinctions, and pick out the most relevant features of exploitation.

First, ‘exploitation’ can be used in a moral or a non‐moral sense. In the non‐moral sense, it just means ‘use’ (as in ‘exploiting an opportunity’). As Feinberg puts it:

To exploit something, in the most general sense, is simply to put it to use, not waste it, take advantage of it.^11^

Here, though, I am interested only in the moral sense of ‘exploitation’, as are most exploitation‐critics of international commercial surrogacy. When used in this sense, the presumption is that something's being exploitative is a defeasible moral reason not to do it. In other words, exploitativeness is a wrong‐making feature of actions.^12^

Such reasons can, however, be overridden by others, so not all instances of exploitation are wrong all things considered. Consider the following example. Imagine that it is established that 80% of trade between the global North and South is exploitative. Imagine too: (a) that it is not practically possible in the short‐term to make it non‐exploitative; and (b) that stopping North‐South trade would further impoverish those in the South and lead to billions of deaths. In such a case it could reasonably be argued that allowing international trade to continue (albeit with some efforts to reduce exploitation) is the right thing to do despite its being exploitative. For, in this case, welfare considerations outweigh the desirability of avoiding exploitation. Welfare, however, need not always outweigh exploitation and there are possible cases in which it is right to avoid a course of action, even a welfare‐maximising course of action, in order to avoid exploitation: for example, we might decide to sacrifice a little beneficial international economic growth in order to prevent markets from becoming considerably more exploitative, or it may sometimes be right to deprive an individual of a small benefit in order to prevent her from being exploited.

Within this moral sense of ‘exploitation’ there is, then, a further distinction between making wrongful use of, and taking unfair advantage of.^13^ The first of these is something akin to ‘commodification’ or ‘objectification’ or ‘treating a person as a mere means’. Such claims are sometimes applied to surrogacy but, if only for terminological clarity, it is best not to use the language of exploitation if commodification or objectification are the main concerns (unless of course the claim is that the practice exploits in *both* senses of ‘exploitation’).^14^

My focus here therefore is on *unfair advantage exploitation*. As I have argued elsewhere, the two key components of an exploitative arrangement (in this sense of ‘exploitation’) are:

(a)  that the exploited person derives (or is at risk of deriving) an unfairly low level of benefit and/or suffers an unfairly high level of cost or harm; and
(b)  that the exploited person's consent to the arrangement is defective or invalid.^15^

Of these, (a) is the most intuitive and obvious. For in standard cases of exploitation, where workers are underpaid, overworked, subjected to discomfort or pain, or endangered by unscrupulous bosses, a fundamental aspect of worker‐exploitation is the fact that they ought to be paid more, ought to be worked less hard, or ought to have better working conditions. And if all such complaints were dealt with then, in most cases, the exploitation would have been vanquished; it would be hard to claim that a worker was exploited whilst also maintaining that she was fairly paid and well treated in all respects.

(b), the point about consent, is less obvious and is more contentious. My suggestion is that exploitation normally occurs only where there is a defect in consent. Against this, someone might claim that exploitation can take place even when valid consent is present. For example, unpaid interns, junior academic faculty, nurses in towns with just one hospital etc. might be exploited even though their consent to take on these positions is voluntary and well informed. There are though reasons for preferring the view that defective consent is a key element in (at least nearly) all cases of exploitation.

The main one is that cases in which there is a worry about unfair distribution and yet entirely valid consent tend to look more like instances of bad luck or generosity or negligence on the part of the putative exploitation‐victim, than genuine cases of exploitation. Let's say that I want to sell you my car. I ask for £10,000 which is close to the market value; you offer me £3,000, which is unreasonably low, but I accept. Whether this is exploitation depends very much on the *reasons why* I chose to accept such a poor offer. If I want to do you a favour, or am rich and do not care about the money, or can't be bothered to find out what the market value is, or just want a quick sale, then the chances are that this is not a case of my having been exploited in the moral sense of that term. If, however, I agree to the sale because you misled me, or because I'm suffering from mental illness, or because your brother threatened me, then this does look like exploitation, but only because any one of these could be a consent‐invalidating factor. So it seems that, when we look at concrete examples, the reasons for labelling a transaction as exploitative are always reasons for being suspicious about the quality of the consent. And indeed it would be hard to understand *why* someone would willingly sign up to an exploitatively ‘bad deal’ unless his or her ability to consent/refuse was compromised in some way.^16^

This also applies to cases like those mentioned above. If a nurse, for example, takes a job in a one‐hospital town voluntarily and on the basis of good information because she believes, all things considered, that that is her best option (perhaps she does not want to leave town, or to commute) then (on the view presented here) she is not exploited (in the relevant sense) even if she is underpaid. However, she may nonetheless be being treated *unfairly*, because not paying someone a fair wage is an independent moral complaint, with or without exploitation.

## 3. Pay and Conditions

It is important to distinguish ‘standard’ from ‘special’ complaints about surrogates' exploitative pay and conditions. Standard ones are essentially the same as ones about the plight of other exploited workers (such as Indian and Nepalese migrant workers in Qatar or the UAE): low pay or no pay; insufficient regard for health and safety; and human rights violations such as imprisonment and physical abuse.^17^ Special ones are generated by the nature of surrogacy such as the fact that it allegedly constitutes baby selling. The term ‘standard’ is not meant to trivialise the complaints. They are only ‘standard’ inasmuch as they are structurally the same as the problems faced by many other workers. They may nonetheless be serious.

### 3.1. ‘Standard’ Arguments

There does seem reason to believe that ‘standard’ concerns about pay and conditions apply to some international surrogacy agreements. Perhaps the most obvious issue is how little some are paid, with their fees being as low as 10% of that commanded by American surrogates. Alison Bailey tells us that:

The entire surrogacy process in the United States can cost between $40,000 and $150,000. Surrogate mothers receive between $20,000 and $30,000 of this sum. In India the complete medical procedure, surrogate's fee, airline tickets, and hotel stay for two trips to India costs around $25,000, but prices can go as low as $12,000. Of that total cost, Indian women are paid between $2,000 and $10,000 for their services.^18^

Also relevant to the exploitation question, as Françoise Baylis notes, is the fact that the very thing that attracts some Western commissioning couples to India is lower cost:

… with specific reference to commercial contract pregnancy in India, the distribution of benefit and harm between contracting couples and reproductive labourers arguably is unjust, insofar as the contracting couples specifically aim to secure increased benefits for themselves at the expense of increased harms to the gestating women.^19^

These consumers of surrogacy services are exploiting Indian poverty: keeping their own costs down by taking advantage of the position of Indian women.

What would count as fair pay is of course notoriously hard to work out. ‘Equal pay for equal work’ is on the face of it attractive, but is it obviously the case that Indian and American surrogates (or workers generally) should receive exactly the same in cash terms given cost‐of‐living differences and the presence or absence of other options? Inflating all developing world wages to the levels of Europe or North America seems neither desirable nor feasible, and (even if it could be implemented) would harm people living in the developing world by pricing them out of the market. On the other hand, do we want to say that fair wage levels should be driven entirely by local standards? If so, then arguably many Indian surrogates are already very well paid. For example, Baylis mentions that rural Indian women stand to gain the equivalent of ten years' salary by becoming surrogates, while a 2012 report in *The Lancet* claims that ‘Indian surrogates earn between $5000 and $7000’ and describes this as ‘an enormous sum for women would normally only earn about $300 a year’.^20^

What we can tentatively conclude then is that, while the details vary from case to case, there is at least an issue about some Indian surrogates being underpaid — although, as just noted, establishing what wage levels are appropriate and fair is a tricky issue across the board, not just for surrogacy.

A second consideration is how surrogates are treated in other (non‐financial) ways. Are they adequately informed at the outset? What conditions must they live under during pregnancy? How is their healthcare managed? What rights do they have at and after birth, including rights to contact with the child? Depending on the answers to such questions, there could be a concern, not only about pay, but also about conditions. And it does seem that there are grounds for concern.

Vida Panitch, for example, reports that some Indian surrogates are ‘housed in clinical compounds where all of their meals and activities are monitored, and from which they are not permitted to leave until delivery’.^21^ And a striking example is the use of Caesarean sections (termed ‘the scissor’ by many Indian surrogates) reported by Amrita Pande. She states:

… surrogates are subjected to another form of medical intervention: caesarean sections. Only two surrogates in th[e] study had natural, vaginal deliveries. This is partly to accommodate the scheduling needs of the intended parents (especially international clients) and the scheduling needs of the clinic.^22^

Pande quotes Razia, a 25‐year‐old surrogate and mother of two, who says:

I delivered yesterday — it was a scissor. My first two children were normal delivery at home. I have never come to a hospital before. Not even when I fell sick with typhoid! I was very scared when they told me I need a scissor. I am very scared of blood and injections.^23^

While Caesarean sections are relatively common even outside the context of surrogacy, there are well‐known risks, including infection of the wound, endometriosis, thrombosis in the legs, excess bleeding, and damage to the bladder or ureter. So to subject surrogates to these risks, not to mention the pain and discomfort, must be a source of concern. This is especially so when the surrogate herself is reluctant and the primary reason for subjecting her to a Caesarean is the scheduling convenience of the commissioning couple or clinic, and when carrying out a Caesarean section for such reasons would not be viewed as good clinical practice in most Western countries.^24^

Another area of potential concern is: what relationship will there be (if any) between the surrogate and the child she carried? If the commissioning parents' and the surrogates' do not have an agreed view on this, one which is acceptable to both parties, conflict and harm may ensue.

So at least some of the ‘standard’ complaints about Indian surrogates' exploitative pay and conditions do seem to be applicable in at least some cases.

What follows from this? In practical and policy terms, standard pay and conditions arguments are vitally important. Surrogates and workers who are maltreated and underpaid are the victims of serious wrongs. However, in principle at least it should be possible to deal with *standard* pay and conditions arguments against international paid surrogacy by enforcing improved pay and conditions. This is something that the Indian government could do, aided and encouraged by the governments of those countries from which the majority of ‘commissioners’ come. Or an ethically defensible international paid surrogacy model could be built along voluntary lines, using a Fair Trade model — something which some ‘commissioning’ parents and healthcare professionals might welcome. Bringing this about will not be easy but, from a purely ethical perspective, this way forward has considerable merit.

An obvious response to ‘standard’ pay and conditions worries then is to seek to improve pay and conditions. This, however, may have negative knock‐on effects on consent. As Baylis puts it:

To increase the payment to Indian women, and further widen the gap between payment for contract pregnancy and payment for other remunerated work, could heighten concerns about the exploitation of economically disadvantaged women in India in relation to the risk of undue inducement.^25^

So, ironically, in seeking to better the position of Indian surrogates, there is a danger that we will further undermine their ability validly to consent (or refuse) to take part in paid surrogacy and will, by allowing ‘undue inducements’, erode their ability to resist. This problem is explored in later sections.

### 3.2. ‘Special’ Arguments — Baby Selling

Special pay and conditions arguments say that there is some feature of surrogacy which means that it is impossible (or nearly impossible) fairly to pay a surrogate. In this subsection, for reasons of space and focus and because this argument is prevalent in the literature, I shall concentrate just on the suggestion that paid surrogacy cannot be fairly rewarded because it is baby selling. There are, however, other possible arguments that one could make: for example, arguments asserting that there is some special feature of gestation that makes fair reward difficult or impossible.

The central claim is that no amount of money is sufficient to compensate for giving up or ‘selling’ a baby. Understood as an *empirical* claim, this is something that *could* be true. For example, psychological evidence *could* reveal that many developing‐world paid surrogates are so damaged by the experience that pretty much no amount of money can restore their welfare levels to what they were before the experience. Pande again writes compellingly of the deep emotional and physical ties that develop between the surrogates and their offspring. She reports that some surrogates had:

… substantial ties with the baby (blood, breast milk) and the effort of gestation makes [them] more attached to the baby than the genetic mother. The reinterpretation of the blood tie and the everyday forms of kinship established between the surrogate and the fetus cannot be dismissed simply as illiterate women's ignorance of Western medicine. The surrogates recognized that they have no genetic connection with the baby, but nonetheless emphasized the (stronger) ties they had with the baby because of shared substances, namely blood and (sometimes) breast milk.^26^

In the light of these ties if would be surprising if at least some surrogates didn't suffer from significant emotional trauma on giving up ‘their’ babies: even if, all things considered, that is what they regard as the best option.

Against this, however, it is worth noting:

(a)  that some surrogates get the equivalent of ten years' salary and, against a backdrop of extreme poverty, that is liable to have a significant positive effect on welfare, one which — at least in many cases — may be sufficient to counterbalance the abovementioned emotional harm;^27^
(b)  that presumably most of those surrogates who consent to paid surrogacy arrangements at least *believe* that they will be better off with than without the arrangement (although of course they might all be mistaken).

The suggestion that no amount of money is sufficient to compensate for ‘selling’ a baby could alternatively be understood as a ‘deeper’ philosophical claim: that some things are not of a kind that can be compensated or rewarded financially. A leading candidate is the baby itself and, as Kimberley Krawiec notes:

Few proposals generate the moral outrage engendered by a suggestion that babies — or, more accurately but less vividly, parental rights — should be traded on the open market. More than anything else, baby selling flies in the face of our deeply held convictions that some items are too priceless to ever be bought and sold.^28^

Whether, however, such thoughts are capable of underpinning an exploitation‐argument specifically against international commercial surrogacy is far from clear.

First, the suggestion that paid surrogacy is baby selling is questionable. This applies especially where there is a genetic link between one or even both commissioning parents and the child. For, in these cases, the commissioning parents have as much of a ‘biological stake’ in the child as the birth mother and (even if gestation ‘trumps’ genetics) can reasonably claim that their situation is quite unlike that of an unrelated person who simply buys a stranger's baby. Even where there is no genetic link, one might still argue that the commissioning couple have a morally transformative causal role in bringing the child into existence.^29^ Furthermore, since the commissioning parents will not normally *own* their children in a full‐blooded sense of ownership (the children will not be slaves) talk of ‘baby selling’ is probably misplaced and misleading.

Second, even if we were to concede that surrogacy is baby selling and that baby selling is wrong, it wouldn't follow from this either that losing (selling) one's baby is not a compensable harm, or that international commercial surrogacy is especially problematic (compared to other forms of paid surrogacy). The latter point is really just another scope‐limitation on the arguments discussed in this article. The ones that interest me here are those which seek to show that *international* commercial surrogacy is *especially* morally problematic and exploitative, whereas the baby selling point will apply (if it applies at all) across the board, not just to international commercial surrogacy, and not just to exploitation concerns.

As regards compensable harm, it may be that adequate compensation is possible even for immoral transactions. Commercial sex is an obvious example here. One view is that commercial sex is wrong: that people should not buy or sell sexual services. It is difficult to see, however, why one could not hold that view *and* also draw a distinction between unpaid, adequately paid, and overpaid providers of sexual services. The same goes for ‘baby selling’. One can disapprove of it whilst also allowing that there may be a compensation threshold (perhaps an extremely high one) above which the person giving up the child is, at least in material or welfare terms, adequately compensated.

So there are two effective responses to the concern that international commercial surrogacy cannot be fairly rewarded because it is baby selling. First, it is far from clear that it really is baby selling. And second, even if it were baby selling, and even on the assumption that baby selling is wrong, adequate compensation may still be possible.

## 4. Consent

I operate here with a fairly standard tripartite account which says that valid consent requires:

Sufficient information (with an opportunity to digest and understand);

Capacity or competence; and

Voluntariness (the absence of distorting or controlling influences, such as coercion or manipulation).

In this section, I briefly review capacity and information issues before looking in more detail at three putative threats to voluntariness: lack of acceptable alternatives, offers that are ‘too good to refuse’, and background poverty and coercion.

### 4.1. Capacity and Information

The main worry under this heading is that many Indian surrogates are uneducated and even illiterate.^30^ This can cause consent problems on at least two fronts. First, if written contracts are involved they will not be able to read them without assistance. As Pande notes:

The surrogacy contract, which lays out the rights of the surrogates, is in English, a language almost none of the surrogates can read.^31^

Second, depending on just how uneducated and in what areas, it may be hard for prospective surrogates to understand what is involved in and what the benefits and risks are. It may also be hard for them to understand their legal position.

These are potentially serious consent problems. How should we respond?

One possible response would be to prohibit international paid surrogacy altogether, or to prohibit it in those countries where lack of education is an issue. Another would be to try to exclude (through a capacity test) individuals whom we fear are insufficiently educated to provide informed consent. A third approach (which could be combined with the second) is to put in place specific procedures aimed at both (i) ensuring that valid consent is obtained prior to any surrogacy agreement (perhaps along the lines of consent to medical treatment or biomedical research), and (ii) maximising people's ability to participate by (for example) using non‐written information (explanatory video perhaps) and by employing staff with strong communication skills to help explain things.

Given the existence of the second and third options, complete prohibition on the basis of capacity or information concerns seems unjustified. If we are worried about some surrogates being insufficiently educated to give proper consent then it should be possible to filter prospective surrogates and to choose only those best able to consent. One downside of this approach is that we may be excluding from paid surrogacy those women who need the money most (the least educated). However, such women would be excluded anyway if we sought to stop international commercial surrogacy altogether and so at least this approach is no worse than that.

It is also worth noting that using a capacity test is something that is justifiable *in all countries*, not just developing world ones, in order to ensure that only those women who are competent to give informed consent become involved in surrogacy arrangements. Economically disadvantage countries are not therefore being singled out for especially negative treatment in this respect; it is just that, in those countries with less effective education systems, more women will fail the test.

### 4.2. Lack of Acceptable Alternatives

This can be dealt with fairly quickly. Lack of acceptable alternatives, on its own (when not caused by coercion or another consent‐invalidating behaviour) is not sufficient to generate a serious consent problem. The decisive counter‐example here is consent to essential life‐saving medical treatment. If my surgeon says ‘if I don't operate then I'm afraid you'll die within 24 hours’, the starkness of that choice does not mean that I cannot validly consent. What is more, it would be more than a little odd to say that operations should not go ahead in such circumstances because of the irresistibility of the surgeon's offer to operate. For it is irresistible precisely because it is so good for me!^32^

### 4.3. Offers That Are ‘Too Good to Resist’

This takes us straight onto the idea (raised by Baylis earlier) that international commercial surrogacy could become ‘too tempting’ for (for example) Indian women if the pay and conditions were improved in an attempt to prevent exploitation. The problem is that paid surrogacy would then be made *even more* attractive than it is now for people in adverse economic circumstances. Thus, women's ability to resist paid surrogacy may be eroded. Let's say that we were to offer prospective surrogates in the developing world similar contractual conditions to American surrogates, along with perhaps half the pay that an American would expect — call this *the improved package*. The worry then is that *the improved package* is *too good* as far as voluntary consent is concerned; it is an offer that prospective surrogates ‘cannot refuse’.

Janet Radcliffe Richards argues that this line of thinking is flawed (and would remain so even if the amount of money were much higher) and that attractive offers in general are ones that can be voluntarily accepted. According to her, one major source of the confusion here is ‘an equivocation between wanting something *in itself* and wanting *all things considered* a package that contains it’.^33^ Thus people tend to say things like — ‘she didn't *really* want to be a surrogate; she *only* did it because she needed the money’ — and this makes it sound as if the decision to donate was somehow forced or involuntary. On Radcliffe Richards' view, we need to distinguish between the following claims:

(a)  If it weren't for the prospect of payment she wouldn't have become a surrogate; that is, considered *in itself*, or *on its own*, surrogacy is not a desirable option for the woman.
(b)  *All things considered*, she prefers being a surrogate and getting the money to not being a surrogate and not getting the money.

Provided that the second of these is true (and if it weren't then she would not have become a paid surrogate in the first place presumably) the decision to donate in return for money can be voluntary and validly consensual. For the woman is still *all things considered* doing what she wants most:

If you dislike the idea of parting with your kidney, but are willing to do it in return for enough money to start a business or send your children to school, you have already decided that doing without the school or the business is *worse* than doing without the kidney. It would be extraordinarily perverse for anyone to claim, on the basis of a concern for voluntariness, that because you disliked one element of the package your consent should be declared invalid, and that you should be left in a situation whose elements you liked even less. And, of course, our criteria for valid consent obviously imply nothing of the sort, or they would prevent our accepting dreary jobs in return for good salaries, or selling anything that we did not positively want to get rid of.^34^

As Radcliffe Richards notes, it would be an odd position to say that while valid consent can be given to a slightly attractive offer, very attractive offers must be withheld because they can't be validly consented to. Her position is broadly correct (and the life‐saving medical treatment example is also telling here) although there may be additional special factors relating to money and poverty that need to be kept in mind too. First, desperation for basic goods (such as food, shelter, and warmth) may make people liable to *non‐autonomously or irrationally* accept low‐quality offers — and to see offers as better than they really are. In the context of international paid surrogacy this can (up to a point) be dealt with by the proposal to ensure that pay and conditions are fair. However, there still could be a residual consent worry that surrogates are overestimating the quality of even *the improved package*. Second, people (especially the poor) are perhaps liable to ignore the diminishing marginal utility of money. Thus, they may (for example) falsely believe that having an extra $50,000 is ten times better for them *in welfare terms* than having $5,000. Where this occurs, it might compromise the capacity/information aspect of consent.

### 4.4. Background Poverty and Coercion

Lack of alternatives *per se* is not sufficient to render consent invalid. So why should background poverty, and more generally lack of economic opportunity, be any different? Isn't this just an instance of lack of alternatives? Sometimes it is. But I suggest that there is an important distinction between situations in which a person's lack of options is caused by brute luck and ones in which it is caused by the coercive actions or omissions of others. For only in the latter case can this (directly) invalidate consent.

The suggestion that whether a person's consent is defective can turn on whether or not their constrained option set was or was not caused by other people's coercive behaviour may strike some people as odd. For either way surely the situation of the consenter is the same, from her point of view, and indeed she may not even know what the causes of her desperate situation are.

Consider this case:

If you have cancer, with the choice between risking its unchecked progression and putting up with pretty nasty treatments, nobody would think of arguing that the narrow range of options made your consent to treatment invalid.^35^

Radcliffe Richards rightly again makes the point that mere lack of options does not invalidate consent. But now consider a different case: one in which *an assailant threatens to kill you unless you ‘donate’ your kidney to his sister*. Both this case and Radcliffe Richards' have this feature in common: the consenter's options are severely constrained. But only in the assailant case would we want to cast doubt upon the validity of the consent and to say that the decision is coerced. This is precisely because the lack of options is brought about by the wrongful coercive behaviour of a third party. This should be no surprise really because valid consent (more so than mere consent) is a richly ethical concept.

It should also be noted that consent can be invalidated by the coercive behaviour of someone other than the person seeking consent.^36^ In the kidney case just mentioned, the consent may be invalid even if given to an *unsuspecting* transplant surgeon; the transplant surgeon does not need to be part of the conspiracy, or even to know about it, for the consent to be defective. Thus, from the consent‐taker's perspective, what *looks like* a valid consent may in reality be invalid because of undetected external coercion. There are special issues in this case about the extent to which the surgeon is responsible for checking that there is no ‘third party’ coercion and about the extent to which we should hold unknowing healthcare professionals responsible in such situations. But returning to the more fundamental point, there seems no reason here to think that a patient's consent to organ donation, given to an unwitting surgeon, cannot be defective as a result of another person's threats. As we will see, this point is potentially important in the case of international surrogacy because, if the source of the consent problem is background poverty, this does not necessarily have much to do with the actions of those seeking women's consent to become surrogates. So if there is coercion in operation it is ‘third party’ coercion, not coercion by the commissioning parents or the surrogacy agency. (This is not to say, of course, that direct coercion by those organising international surrogacy does not happen — merely that this is not the subject of the present discussion.)

Turning back to the case at hand, it is plausible to suppose that at least some Indian surrogates' background poverty is not morally neutral (that it has been caused by the wrongful actions or omissions of others) and, more specifically, that it is an instance of coercion — or at least it is structurally so similar to coercion that it has coercion's consent‐invalidating characteristics. The form of the threat here is that unless the woman accepts a low‐quality job of one kind or another (surrogacy being one of the ‘job options’) then she and her family will be faced with destitution. This is arguably coercive or at least quasi‐coercive, rather than merely a case of brute luck because (let's assume) the government is knowingly creating the threat of starvation by failing to deliver on its obligations: failing to provide adequate welfare benefits for those not working perhaps, or failing to implement policies that would encourage the development of more and better jobs.^37^

As regards the question of whether the Indian government (or any other government for that matter) is in fact behaving coercively, this is of course not something that can be answered properly here. It is a question that raises very complex economic issues as well as ones in political philosophy. With that caveat in place, one very general reason for thinking that the background poverty might not be morally neutral is the huge disparity in wealth and income between rich and poor countries. For example, according to the World Bank, per capita GDP in the Canada and the United States is presently more than US$50,000, whereas the figure for India is around US$1,500 (and even for Ukraine, another popular surrogacy destination — at least before recent political unrest — the figure is less than US$4,000).^38^ Given this gap anyone with even the slightest egalitarian instinct may well conclude that there are strong obligations to alleviate poverty in places like India.

There may also be more specific reasons to blame governments. For example, Baylis tells us that:

Since the 1990s, the Indian government has focused on GDP growth at the expense of issues of social justice and equality. It has actively encouraged reproductive travel to India as a way to grow the economy without simultaneously (i) addressing the background social, material, and political conditions against which women in grinding poverty make choices about employment (and in some cases survival) and without (ii) developing a robust regulatory framework for reproductive travel in order to protect the health and well‐being of Indian women.^39^

So, if one believes that Indian women's poverty is at least partly the result of coercive or quasi‐coercive acts or omissions of governments, and quite possibly also of businesses and individuals, that does provide grounds for thinking of their consents as defective.

## 5. Ethical and Policy Implications

Many surrogates' adverse background conditions then seem to be coercive or quasi‐coercive. What follows from this?

One option is to say that international paid surrogacy is unethical and should be discouraged or prohibited, even if pay and conditions for surrogates were improved, because the chances of consent being defective are too high.^40^ However, even if these concerns about consent are legitimate (which they probably are) there are reasons for not adopting a wholly negative or prohibitive stance towards international paid surrogacy.

Most obviously, it is important to consider the welfare‐implications of prohibiting international commercial surrogacy on several different groups. First, there are the surrogates themselves (and their families). If their plight is as desperate as exploitation‐critics suppose, then there is every chance that prohibition will substantially harm women who would otherwise have earned money from surrogacy. Since the lack of decent alternatives is a key part of the exploitation‐critique, we can only assume that these women will not readily find equally well‐paid alternatives. Second, there are prospective commissioning parents. Prohibiting international commercial surrogacy would prevent some of these people from having their own children. Others may be tempted to ‘go underground’ and to enter into unlawful surrogacy arrangements, either within their own country or by crossing borders. Third, prohibiting international commercial surrogacy may result in, and indeed is designed to result in, many possible future children not being born (the children who would have been born through international surrogacy) most of whom would have had worthwhile lives. While it would be going too far to say that this group (the merely possible future children) would be harmed, governments should, at the very least, be cautious about using legal prohibition to prevent people from coming to exist in cases where: (a) the child would be wanted by its parents; (b) the parents can afford to take care of the child and are capable of providing him or her with a decent home; and (c) the child would have a reasonable quality of life.

So given that the immediate effects of banning international commercial surrogacy would include harm to prospective surrogates, harm to prospective commissioning parents, and the non‐existence of future persons who would otherwise have had worthwhile lives, there does seems to be a powerful argument for allowing international commercial surrogacy *despite* any qualms about the quality of Indian women's consents. This is a straightforward balancing position with other considerations being taken to outweigh concerns about defective consent.

This balancing argument is bolstered by a point made by Janet Radcliffe Richards in one of her discussions of organ sale. She argues that there is something fundamentally wrongheaded about preventing a person from accessing an option that would be hugely beneficial to her when the supposed justification for this is that the person's consent is defective. To illustrate, she asks us to consider a case in which a family's child is kidnapped and a ransom demanded. This is a clear case of coercion. It is therefore reasonable to suppose that the family's consent to any transaction with the kidnappers (such as handing over money in exchange for a release) would be insufficiently involuntary and so defective. Should we, Radcliffe Richards asks, therefore prevent the family from transacting with the kidnappers?^41^

Suppose the police appeared on the kidnapping scene and prevented you from signing the document [*a contract with the kidnappers*] perhaps with the outcome that your child was shot. They might have good public policy reasons for this … but it would be preposterous for them to claim that they were doing it because the consent you were trying to give would be invalid … the whole point of declaring invalidity is to protect the alleged consenter, and here the police would actually be compounding the wrong done to you by constricting still further the range of options already constricted by the kidnapper.^42^

Applying this line of thinking to surrogacy, we might say that, even if impoverished women's consents to be surrogates are defective, we nonetheless have good reason to accept their consents (especially as the defect is one that has little to do with any defects in surrogacy practice, but is rather a structural flaw that would afflict almost any paid activity that these women agreed to). To do otherwise would be to restrict still further their range of options, and also to harm them if paid surrogacy really is their best option. The point of insisting on valid consent is to protect the consenter — and insisting on it where this will be clearly harmful and option‐constraining would be perverse.

A second related reason for not prohibiting international paid surrogacy is that doing so may (as has already been mentioned) drive it underground. Michael Freeman, as far back as the 1990s, made this point forcefully in response to the *Brazier Report*. That report proposed a ban on commercial surrogacy in the UK: specifically that payment ‘should cover only genuine expenses associated with the pregnancy’ and that ‘additional payments should be prohibited in order to prevent surrogacy arrangements being entered into for financial benefit’.^43^ Freeman concludes:

If Parliament agrees with Brazier and denies women the opportunity to be financially rewarded for surrogacy services, an unregulated surrogacy with all the evils attendant upon such underground activities will emerge.^44^

Freeman's prediction that attempts to prevent paid surrogacy in the UK would result in people merely subverting the law, either at home or by going overseas, has proven broadly correct and there is every reason to suppose that a prohibitive approach to international commercial surrogacy would have similar results: that people would seek to bypass it in one way or another. Given that there are very strong desires or needs in play on both sides — that many people desperately want to use surrogacy services, and that many prospective surrogates desperately need the money — the chances of prohibition succeeding are slight. As Freeman puts it:

We cannot stop women exercising their autonomy, nor can we persuade them, that being paid aggravates their exploitation, when common sense tells them the reverse.^45^

If surrogacy is going to take place anyway then it would be better if it occurred ‘above ground’: if it were accepted and regulated rather than being left ‘underground’ or in a legal ‘grey area’. Without acceptance and regulation, various evils may ensue, and indeed such problems occur now in places where paid surrogacy is not accepted and regulated. For example, there are sometimes problems with legal status; children can be left legally parentless and stateless; while it may be impossible for the child's social and genetic parents to become its legal parents.^46^ Also, in common with other areas of prohibition (illegal drugs, pornography, sex work, etc.) banning surrogacy may provide opportunities for organised crime to become involved, with all the negative side‐effects that entails.

A third reason why prohibition may not be the best option is that there is a less draconian alternative: improving paid surrogacy practices in the commissioning parents' home countries. This article addresses concerns about consent and exploitation in *international* commercial surrogacy arrangements, specifically those where the commissioning parents come from relatively affluent countries and the surrogates are relatively poor financially (such as many Indian surrogates). I have conceded that such situations do give cause for concern. One way of dealing with such concerns though — an approach which aims to reduce the prevalence of international surrogacy, whilst stopping short of prohibition — is to make surrogacy (especially paid surrogacy) work better in the prospective parents' home countries. This is a possible solution because many Western parents turn to international surrogacy (which is often inconvenient and risky) mainly because domestic surrogacy is either banned or otherwise legally restricted at home. Natalie Gamble, for example, tells us that:

British parents *struggling to find an altruistic surrogate mother in the UK (not least because of the legal restrictions here)* are attracted abroad to countries where there is ready availability of willing host surrogate mothers, many of them offering their services on a commercial basis. Eastern Europe, the USA and India are all popular destinations.^47^

What would making surrogacy ‘work better’ in the prospective parents' home countries amount to? This is a huge question that would require another paper to answer. However, key elements include: (a) not prohibiting, or unduly restricting, payment to surrogates (in order to increase ‘domestic supply’); (b) ensuring that there is legal certainly and clarity about who gets what parental rights and responsibilities; and (c) some degree of ‘enforceability’ of surrogacy contracts (although preferably with courts having discretion to override these, for example to protect the welfare of the child).^48^

Finally, when considering whether or not to prohibit international commercial surrogacy, we need to consider the question of consistency. As various commentators note, the consent worry about adverse background conditions applies equally to any paid activities and hence to surrogates' alternative sources of income too.^49^ Therefore, one might say that it is inconsistent and unfair to attempt to prevent international paid surrogacy, whilst still allowing many other forms of economic activity to continue. Baylis (while criticising this line of argument) outlines it clearly as follows:

No doubt some will mount a *reductio ad absurdum* response to this facet of my argument against transnational commercial contract pregnancy in India, by pointing to a range of exploitative employment practices in low‐income countries that the world not only tolerates, but encourages. ‘Your argument,’ they will say, requires you to reject all manner of unfair advantage exploitation that undergirds the modern world of international capitalism. ‘You’ cannot condemn transnational commercial contract pregnancy in India without at the same time condemning analogously exploitative practices such as child labour in sweatshops, dangerous work decommissioning nuclear ships, or underpaid work crushing glass in factories.^50^

If we are merely discussing *the ethics of* international commercial surrogacy then this consistency argument is vitally important. It is axiomatic that, other things being equal, a coherent ethical position is preferable to one which is inconsistent. And so, for the reasons that Baylis cites, it looks as if we must conclude that international commercial surrogacy is quite possibly no worse than many other aspects of international capitalism, and indeed it may be *relatively* benign compared to some of the worst construction or factory jobs.

When, however, we turn from ethics to policy, consistency considerations play out slightly differently because we are not starting with a ‘blank page’. The prohibition of illegal drugs is a fairly standard case in point. Arguably, the case for banning alcohol and tobacco is pretty strong compared with the case for banning some illegal substances. So consistency considerations might compel us to ban all or ban none. But since alcohol and tobacco use is already entrenched in many countries and, given that prohibiting these immediately would be bound to fail, it may be that a policy approach that deals with them more leniently than the medical evidence suggests they deserve is justified. The justification is that we ban harmful substances *where we can* and *where this will work*. And we do not have to do nothing just because we cannot do everything. Baylis again makes the point well as follows:

I cannot take on international capitalism. Here, I focus my efforts on the considerably more modest project of trying to build a coherent and persuasive argument against one instance of unfair advantage exploitation, namely transnational commercial contract pregnancy in India. My goal is to stop this nascent practice — which involves the exploitation of poor women in low‐income countries by rich and middle‐class women and men — before it is normalized as a legitimate capitalist activity. Other analogously exploitative practices are well entrenched and for this reason are exceedingly difficult (if not impossible) to abolish …^51^

Baylis's position is that we should stop what exploitation we can and the fact that we cannot stop it all is not a reason not to stop new forms emerging.

On a practical and policy level this is a coherent and not entirely unattractive position. However, there are nonetheless potential problems with it. Most importantly, it is a revolutionary view since it *seems in principle to* be committed to the abolition of all the aspects of ‘international capitalism’ that involve exploitation. On this view, the only reason for allowing such practices to continue is that they are ‘exceedingly difficult to abolish’; if it could be abolished, they should be. This is not necessarily a damning objection (some revolutionary views are defensible) but it does at least raise difficult questions. For example, how would the abolition of international capitalism impact on the millions or billions of people whose livelihoods depend on (often exploitative) international trade arrangements? And what socio‐economic arrangements would or should be introduced once international capitalism has been all but abolished? Perhaps these questions can be answered and I don't argue here that they can't; such a claim would be way beyond the scope of this article. I merely note for now that, while Baylis's position *seems* admirably pragmatic, it may merely be postponing or concealing some difficult questions about what the realistic alternatives to ‘international capitalism’ are and about how the desirability of avoiding exploitation is to be weighed against other considerations, notably people's welfare.

That said, I do concede that ethics and policy may, and for the reasons offered by Baylis, come apart a little at this juncture. If we are focussing solely on the *ethics* of international commercial surrogacy, it does seem (at least as far as consent and exploitation are concerned) that it is in no worse a position than many other paid activities. Therefore, our ethical qualms about surrogacy should be viewed as merely as subset of more general concerns about economic injustice and its implications for autonomy (amongst other things). On a *policy* level though it is not possible to reach such a clear and swift conclusion. Merely making the consistency point is not *obviously* sufficient to constitute a compelling argument for treating surrogacy just like everything else.

## 6. Conclusions

There is genuine cause for concern about exploitation in international commercial surrogacy arrangements. Underpinning this is a combination of substandard pay and conditions and defective consent. At least in principle, we can deal with the former by ensuring that surrogates are better paid, that their living conditions are decent during pregnancy, that their health and welfare are taken properly into account when deciding about issues like Caesarean section, and that the terms contained in surrogacy contracts are transparent and not unreasonably onerous. Several commentators, however, have suggested that improving pay and conditions, while perhaps removing the exploitation concern, may exacerbate the consent problem. In particular, improving pay and conditions would make paid surrogacy an even more attractive option against a backdrop of extreme poverty.

In this article, I have rejected some versions of this consent argument but accepted others (with certain qualifications). The one that I accept says that if the surrogates' background poverty is caused by the coercive or quasi‐coercive acts and omissions of other people or institutions (which probably it is, at least in part) then this poverty may degrade the quality of surrogates' consents. To this extent the exploitation‐critique of international commercial surrogacy is a success; for while exploitation can be avoided by addressing pay and conditions, this can only be done at the cost of exacerbating consent problems.

What are the implications of this? While ethically there is something to be concerned about here, paid surrogacy is likely to be in no worse a position than many other exploitative commercial transactions which take place against a backdrop of global inequality and constrained options. Hence, there is little reason to single it out for special condemnation. While on a policy level, the case for prohibiting international commercial surrogacy remains weak, even in the light of these concerns about consent. Some reasons for this conclusion are pragmatic: the wellbeing of surrogates and the fear that prohibition will merely drive commercial surrogacy underground. Others are more ‘principled’, like Radcliffe Richards' argument that the point of insisting on consent is to protect the consenter and that it would therefore be perverse to insist on a high standard of consent if that meant constricting still further the range of options available to economically disadvantaged women.

If international paid surrogacy is allowed to continue though it is important that state actors and international bodies, such as the Hague Conference on Private International Law, devise agreements and regulations that (amongst other things) seek to ensure that surrogates have a fair minimum wage, that contractual terms are transparent and not unduly onerous, that living conditions and healthcare for surrogates are decent, and that the legal status of any children created is clear.

But while wanting to attain these high standards is laudable, any attempt to do so faces formidable challenges. Some of these are ‘conceptual’. *What counts as a fair minimum wage? What does it take for a contractual condition to be unduly onerous? What level of healthcare is ‘decent'?* Others are political. *Can we get countries with divergent systems of family law to sign up to and then operationalize an international convention on international surrogacy?* On this point perhaps the existence of the Hague Children's Conventions give us some reason for cautious optimism. Hannah Baker writes:

The implementation of the modern Hague Children's Conventions has been described as a continuing, progressive or incremental process of improvement. Undoubtedly the same will be true if there is ever an instrument on international surrogacy, and this must be at the forefront of our minds: the mere creation of a convention would only be the first step (albeit, perhaps, a ‘giant leap’).^52^

Finally, as Baker suggests, the regulation of international surrogacy faces substantial practical difficulties. Even with sound regulations ‘on the books’, individuals and agencies will no doubt attempt to subvert or evade what rules exist. There is the ever‐present danger of corruption. And there would be a risk of regulatory overreach and excessive bureaucracy, which might *unduly* restrict markets in surrogacy services with the ultimate outcome being unwarranted suffering for childless couples, and the non‐existence of children who — if born — would have had lives well worth living. The scale of these implementation problems should not be underestimated but, in defence of a regulated international surrogacy sector, I note again that similar problems would afflict the main alternative, prohibition. In particular, prohibition (in this area as in others) drives otherwise law‐abiding citizens to become actors in ‘black’ or ‘grey’ markets, a situation in which they are more vulnerable to exploitation or harm than they otherwise would be. Thus, while a regulated international surrogacy sector is unlikely to work perfectly any time soon, much the same goes for prohibition; hence concerns about enforceability and implementation do not give us reason to be sceptical specifically about the ‘regulated market’ approach.^53^

## Notes

1

Françoise Baylis , ‘Transnational commercial contract pregnancy in India’ in BaylisF. & McLeodC. (eds) Family‐making: Contemporary Ethical Challenges (Oxford: Oxford University Press, 2014), p. 265.

2

Amrita Pande , ‘Transnational commercial surrogacy in India: Gifts for international sisters?’, Reproductive Biomedicine Online 23 (2011): 618–625;2195891610.1016/j.rbmo.2011.07.007 Alan Wertheimer , ‘Exploitation and paid surrogacy’, Denver University Law Review 74 (1996–1997): 1215.16437783 Alan Wertheimer , ‘Two questions about surrogacy and exploitation’, Philosophy and Public Affairs 21,3 (Summer, 1992): 211–239.11652070 Mary Warnock et al., Report of the Committee of Inquiry into Human Fertilisation and Embryology (London: HMSO, 1984).

3

Stephen Wilkinson , Bodies for Sale: Ethics and Exploitation in the Human Body Trade (London: Routledge, 2003), pp. 134–181.

4
Krawiec notes that often ‘surrogates earn a relatively small share of the total price paid by intended parents, often less than the agency fee and typically much less than the total price paid, which includes medical and legal expenses and the agency placement fee’: Kimberley Krawiec , ‘Altruism and intermediation in the market for babies’, Washington and Lee Law Review 66 (2009): 203–257, at p. 244. Arguably, then, agencies may be exploiting both surrogates and commissioning parents.

5
See for example: Amel Alghrani , ‘Surrogacy: “A cautionary tale” — *Re T (a child) (surrogacy: residence order) [2011] EWCH 33 (Fam)*
’, Medical Law Review 20,4 (2012), 631–641;2309745810.1093/medlaw/fws032 Geoff Bennett , ‘Bristol surrogate mum jailed after faked pregnancies scam’, 
*Bristol Post*
 16 June 2013; Steven Morris , ‘Surrogate mother jailed for faking pregnancies’, 
*The Guardian*
 16 June 2014.

6
The recent ‘Baby Gammy’ story is a case in point. See for example: Sonia Allen , ‘Baby Gammy case reveals murky side of commercial surrogacy’, 
*The Conversation*
 4 August 2014 Available at: http://theconversation.com/baby‐gammy‐case‐reveals‐murky‐side‐of‐commercial‐surrogacy‐30081

7
According to COTS (a British surrogacy organisation) ‘98 per cent of arrangements involving COTS members have reached successful conclusions’. Available at: http://www.surrogacy.org.uk/FAQ4.htm

And, according to Alghrani, there have only been (or rather had only been by the Autumn of 2012) five reported British legal cases in which surrogates have refused to relinquish the child. 4 out of 5 of these were cases in which the surrogate's own eggs were used: Alghrani op. cit.

8

Shekhar Bhatia , ‘Revealed: How more and more Britons are paying Indian women to become surrogate mothers’, 
*Daily Telegraph*
, 26 May 2012. This figure was confirmed at a round‐table convened by the Project Group on Assisted Reproduction (PROGAR). Eric Blyth , Marilyn Crawshaw & Olga van den Akker , ‘What are the best interests of the child in international surrogacy?’, 
*Bionews*
 17 February 2014 Available at: http://www.bionews.org.uk/page_397263.asp

9

Richard Ashcroft , ‘Money, consent, and exploitation in research’, The American Journal of Bioethics 1,2 (2001): 62–63 at p. 62.1195189810.1162/152651601300169158

10
For much fuller treatments of exploitation see, for example: Richard Arneson , ‘What's wrong with exploitation?’, Ethics 91,2 (1981): 202–227; Lynn Jansen & Steven Wall , ‘Re‐thinking exploitation: A process‐centred account’, Kennedy Institute of Ethics Journal 23,4, (2013): 381–410;2455207710.1353/ken.2013.0015 ReeveAndrew (ed.), Modern Theories of Exploitation (London: Sage, 1987); Ruth Sample , Exploitation: What It Is and Why It's Wrong (Lanham, MD: Rowman & Littlefield, 2003); Mikhail Valdman , ‘A theory of wrongful exploitation’, Philosophers' Imprint 9,6 (2009): 1–14; Alan Wertheimer , Exploitation (Princeton, NJ: Princeton University Press, 1999); Wilkinson 2003 op. cit.; Alan Wood , ‘Exploitation’, Social Philosophy and Policy 12,2 (1995): 136–158.

11

Joel Feinberg , Harmless Wrongdoing: The Moral Limits of the Criminal Law, vol. 4 (New York: Oxford University Press, 1990), p. 177.

12
This may not apply in certain special contexts, such as just punishment or self‐defence. I doubt however that this is very often relevant to international paid surrogacy and so we can assume that exploitativeness is wrong‐making for the present discussion.

13

John Harris , The Value of Life (London: Routledge, 1990), p. 120. Wilkinson 2003 op. cit., pp. 13–16.

14

Wilkinson 2003 op. cit., pp. 27–56.

15
One side‐issue is whether cases where there is no consent at all, as opposed to those in which consent occurs but is defective, can be cases of exploitation. Elsewhere, I have argued that there does need to be defective consent and that ‘non‐consent’ cases aren't exploitation. I do not however need to rely on or discuss this assumption here because all of the surrogacy cases under consideration are going to be ones where there is consent of some kind ( Wilkinson 2003 op. cit., pp. 72–75).

16
The role of consent is one of the biggest issues in exploitation theory. See note 10 for some of the literature on this.

17
‘Indian migrant workers return home with horror tales from UAE’, 
*The Times of India*
 13 February 2013 Available at: http://articles.timesofindia.indiatimes.com/2013‐02‐13/middle‐east‐news/37078330_1_migrant‐rights‐council‐mrc‐uae‐government; ‘ “Open prison”: Worker deaths cast shadow over Qatar World Cup’, 
*Spiegel Online International*
 1 October 2013 Available at: http://www.spiegel.de/international/world/migrant‐workers‐dying‐in‐qatar‐ahead‐of‐2022‐world‐cup‐a‐925397.html; ‘Revealed: Qatar's World Cup “slaves” ’, 
*The Guardian*
 25 September 2013 Available at: http://www.theguardian.com/world/2013/sep/25/revealed‐qatars‐world‐cup‐slaves

18

Alison Bailey , ‘Reconceiving surrogacy: Toward a reproductive justice account of Indian surrogacy’, Hypatia 26,4 (2011): 714–741, at p. 718. See also: Vida Panitch , ‘Surrogate tourism and reproductive rights’, Hypatia 28,2 (2013): 274–289.

19

Baylis op. cit., p. 271.

20

Baylis op. cit., p. 271; Priya Shetty , ‘India's unregulated surrogacy industry’, The Lancet 380,10 November (2012): 1633–1634.10.1016/s0140-6736(12)61933-323162822

21

Panitch , op. cit., p. 282.

22

Amrita Pande , Wombs in Labor: Transnational Commercial Surrogacy in India (New York: Columbia University Press, 2014), p. 122.

23
Ibid.

24

NICE (National Institute for Health and Clinical Excellence, UK) , ‘Caesarian section: NICE clinical guideline’, November 2011 Available at: http://guidance.nice.org.uk/CG132/Guidance/pdf/English

25

Baylis op. cit., p. 271.

26

Amrita Pande , ‘ “It may be her eggs but it's my blood”: Surrogates and everyday forms of kinship in India’, Qualitative Sociology 32 (2009): 379–397, at p. 384.

27

Shetty op. cit.

28

Krawiec op. cit., p. 204.

29
On these grounds, some people have argued the commissioning parents should be thought of as more like people using assisted reproduction, than like adoptive parents. See for example: Natalie Gamble , ‘A better legal framework for UK surrogacy?’, in GolombokSusan et al. (eds) Regulating Reproductive Donation (Cambridge: Cambridge University Press, 2015 in press).

30

Panitch op. cit., p. 283.

31

Amrita Pande , ‘Commercial surrogacy in India: Manufacturing a perfect mother‐ worker’, Signs 35 (2010): 969–992, at p. 976.

32

Martin Wilkinson & Andrew Moore , ‘Inducement in research’, Bioethics 11,5 (1997): 373–389, at p. 377.1165511710.1111/1467-8519.00078

33

J. Radcliffe Richards , ‘Consent with inducements: The case of body parts and services’ in MillerF. & WertheimerA (eds) The Ethics of Consent: Theory and Practice (Oxford: Oxford University Press, 2006). pp. 281–304, at p. 290.

34
Ibid., p. 290. See also S. Wilkinson , ‘Biomedical research and the paid exploitation of human tissue’, Genomics, Society, & Policy 1 (2005): 27–40.

35

Radcliffe Richards op. cit., p. 291.

36

Wilkinson 2003 op. cit., pp. 82–98.

37
Quasi‐coercive situations (in this context) are those which, while not technically instances of coercion, are sufficiently similar to coercion in morally relevant respects to share its consent‐invalidating properties.

38
http://www.worldbank.org/

39

Baylis op. cit., pp. 273–274.

40
Baylis, for example, concludes that international commercial surrogacy ‘fails morally and should be abolished’ ( Baylis op. cit., p. 283).

41
It is important to note, as Radcliffe Richards does, that there may be other reasons — i.e. ones not to do with invalid consent — to prevent the family from paying the ransom. Similarly, there may be other reasons not to allow international commercial surrogacy.

42

Radcliffe Richards op. cit., p. 294.

43

Margaret Brazier , Alastair Campbell & Susan Golombok , 
*Surrogacy: Review for Ministers of the Current Arrangements for Payment and Regulation*
, CM 4068 (London: Department of Health, 1998), p. 71.10.1093/humupd/3.6.6239584949

44

Michael Freeman , ‘Does surrogacy have a future after Brazier?’, Medical Law Review 7 (1999): 1–20, at p. 20.1165802410.1093/medlaw/7.1.1

45

Freeman op. cit., p. 10.

46

Natalie Gamble , ‘Crossing the line: the legal and ethical problems of foreign surrogacy’, RBM Online 19,2 (2009): 151–152.1971254610.1016/s1472-6483(10)60064-8

47

Gamble 2009 op. cit., p. 151, my emphasis.

48
For more detailed discussions of this see Gamble 2015 in press op. cit.

49

Wilkinson 2003 op. cit., pp. 134–181.

50

Baylis op. cit., p. 274.

51

Baylis op. cit., p. 275.

52

Hannah Baker , ‘A possible future instrument on international surrogacy arrangements: Are there “lessons” to be learnt from the 1993 Hague Intercountry Adoption Convention?’ in TrimmingsK. & BeaumontP. (eds) International Surrogacy Arrangements: Legal Regulation at the International Level (Oxford: Hart Publishing, 2013), pp. 411–426, at p. 426.

53
I would like to thank the following people for helpful comments and questions: Françoise Baylis, Andrew Botterell, Monique Deveaux, Kimberly Krawiec, Patti Lenard, Carolyn Macleod, Christine Overall, Amrita Pande, Vida Panitch, Vardit Ravitsky, Christine Straehle, Martin Wilkinson, and two anonymous referees for the *Journal of Applied Philosophy*.

